# Association between *IL-37* gene polymorphisms and risk of HBV-related liver disease in a Saudi Arabian population

**DOI:** 10.1038/s41598-019-42808-4

**Published:** 2019-05-09

**Authors:** Mashael R. Al-Anazi, Sabine Matou-Nasri, Arwa A. Al-Qahtani, Jahad Alghamdi, Ayman A. Abdo, Faisal M. Sanai, Waleed K. Al-Hamoudi, Khalid A. Alswat, Hamad I. Al-Ashgar, Mohammed Q. Khan, Ali Albenmousa, Monis B. Shamsi, Salah K. Alanazi, Damian Dela Cruz, Marie Fe F. Bohol, Mohammed N. Al-Ahdal, Ahmed A. Al-Qahtani

**Affiliations:** 1 Department of Infection and Immunity, Research Center, King Faisal Specialist Hospital & Research Center, Riyadh, Saudi Arabia; 2 Medical Genomics Research Department, King Abdullah International Medical Research Center, King Saud bin Abdulaziz University for Health Sciences, Ministry of National Guard Health Affairs, Riyadh, Saudi Arabia; 3 Department of Family Medicine, Prince Mohammed Bin Abdul Aziz Hospital, Riyadh, Saudi Arabia; 4 Section of Gastroenterology, Department of Medicine, College of Medicine, King Saud University, Riyadh, Saudi Arabia; 5 Gastroenterology Unit, Department of Medicine, King Abdulaziz Medical City, Jeddah, Saudi Arabia; 6 Gastroenterology Unit, Department of Medicine, King Faisal Specialist Hospital & Research Center, Riyadh, Saudi Arabia; 7 Department of Gastroenterology, Prince Sultan Medical Military City, Riyadh, Saudi Arabia; 8 Centre for Genetics and Inherited Diseases, College of Medicine, Taibah University, Madinah, Saudi Arabia; 9 Liver Disease Research Center, King Saud University, Riyadh, Saudi Arabia; 10 Department of Microbiology and Immunology, Alfaisal University School of Medicine, Riyadh, Saudi Arabia

**Keywords:** Mutation, Cancer

## Abstract

Interleukin-37 (IL-37) has recently been recognized as a strong anti-inflammatory cytokine having anti-tumor activity against hepatocellular carcinoma (HCC) in hepatitis B virus (HBV)-infected patients. HCC is a typical inflammation-related cancer, and genetic variations within the *IL-37* gene may be associated with the risk of HBV infection. Identification of the allelic patterns that genetically have a high disease risk is essential for the development of preventive diagnostics for HBV-mediated liver disease pathogenesis. In this study, we aimed to investigate the association between single nucleotide polymorphisms (SNPs) within the *IL-37* gene and disease sequelae associated with HBV infection. We genotyped ten *IL-37* SNPs in 1274 patients infected with HBV and 599 healthy controls from a Saudi Arabian population. Among the selected SNPs, two SNPs (rs2723175 and rs2708973) were strongly associated with HBV infection, and six SNPs (rs2723176, rs2723175, rs2723186, rs364030, rs28947200, rs4392270) were associated with HBV clearance, comparing healthy controls and HBV infected-patients respectively. A suggestive association of rs4849133 was identified with active HBV surface antigen (HBsAg) carrier and HBV-related liver disease progression. In conclusion, our findings suggest that variations at the *IL-37* gene may be useful as genetic predictive risk factors for HBV infection and HBV-mediated liver disease progression in the Saudi Arabian population.

## Introduction

Hepatitis B virus (HBV) is a blood-borne virus that specifically infects the liver and triggers immune-mediated liver injury, which may result in cirrhosis or hepatocellular carcinoma (HCC) in extreme cases^1^. HBV is vertically transmitted from infected mothers to their offspring, though it is more frequently acquired through horizontal transmission including contaminated blood transfusion, parenteral routes or via sexual interaction^2,3^. HBV infection affects more than 2 billion people globally, with a high prevalence in Sub-Saharan African and Southeast Asian countries including Saudi Arabia^2,4^. In approximately 90% of cases, HBV infection is acute, and the virus is cleared within 6 months by the natural immune response^5,6^. Most patients chronically infected with HBV are asymptomatic and characterized by the absence of the HB e antigen (HBeAg). The presence of anti-HBe antibodies is associated with largely intact liver tissue^2^. Based on available age-related epidemiological data, in 90% of infants and 50% of young children affected with HBV, the infection becomes chronic and persists for many years^7,8^.

Belonging to the *Hepadnaviridae* family that replicates in human hepatocytes, HBV is an enveloped non-cytopathic virus containing a partially double-stranded viral DNA genome of 3.2-kb length within its core^9,10^. After infecting hepatocytes, HBV releases its genome into the cell host nucleus for viral RNA transcription, DNA replication, and viral protein synthesis including HBV surface antigen (HBsAg). The degree of severity of HBV infection is influenced by several factors such as the age at infection, longer duration of infection, immune status, HBV genotype, high degree of viral mutations, high level of HBV replication, co-infection with hepatitis C or delta virus, or with human immunodeficiency virus (HIV), male gender, environmental factors (e.g., alcohol consumption, smoking and exposure to aflatoxin), and ethnic background^11–16^.

Recent studies provided additional evidence of the pivotal role of inflammation in patients with chronic HBV infection, which may result in cirrhosis following secondary necroinflammation with the eventual progression to HCC^17,18^. Pro-inflammatory mediators, such as interferons and cytokines, are produced after the binding of the HBV core protein to membrane heparin sulfate exposed on the cell surface of human hepatoma cells^19,20^. Interleukin-37 (IL-37), a member of the IL-1 family, is an anti-inflammatory cytokine produced by immune cells and suppresses the production of inflammatory cytokines in several types of disease. It has been shown that IL-37 is capable of reducing the activity of both innate and specific immune responses^21,22^. Zhao *et al*. (2014) showed that decreased expression of IL-37 was correlated with HCC progression^23^, and elevated serum IL-37 levels have been observed in patients infected with HBV and treated with telbivudine^24^. Several studies have shown that there is a significant association between certain genetic variations within the *IL-37* gene and several diseases, including tuberculosis^25,26^, coronary artery disease (CAD)^27^, and autoimmune-based thyroid diseases^28^.

Despite the implementation of anti-HBV immunization programs for newborns, there are still approximately 5,000 new patients diagnosed with HBV infection per year in Saudi Arabia^4,29^. In this study, we investigated the association between *IL-37* SNPs and disease sequelae associated with HBV infection in a Saudi Arabian population.

## Materials and Methods

### Patients

Peripheral blood samples were collected from 1274 patients infected with HBV and 599 normal healthy volunteers of Saudi origin from three major hospitals in Riyadh City, including King Faisal Specialist Hospital and Research Center (KFSHRC), King Khalid University Hospital (KKUH), and Prince Sultan Military Medical City (PSMMC). Written informed consent was obtained from participating individuals, and the study was approved by the institutional review board of the participating hospitals in accordance with the Helsinki Declaration of 1975. The patients were grouped in five categories based on disease severity: group I included patients who cleared HBV (n = 400), group II patients with inactive HBV infection (n = 563), group III patients with active HBV infection (n = 217), group IV patients with HBV-associated cirrhosis (n = 64), and group V patients with cirrhosis diagnosed with HCC (n = 30). Control subjects were characterized by the absence of any known serological marker for HBV.

### TagSNP Selection

The SNP data of the entire *IL-37* gene were downloaded from the 1000 Genomes Project Database (GPD; http://www.internationalgenome.org). All genetic variants with a minor allele frequency ≥ 0.05 and located within the *IL37* genomic region (Chromosome 2: 113,670,548-113,676,459, GRCh37) plus a flanking region of 7 kb were extracted from the 1000 Genome Project – Phase 3^30,31^. The Tagger tool as implemented in Haploview Software (Broad Institute of MIT and Harvard, Cambridge, MA, USA, version 4.2) was used to select tag SNPs that span this genomic region using the pairwise tagging method and an *r*^2^ threshold of 0.8. Of the identified 134 variants in the 1000 GPD, we selected 10 tag SNPs that captures 103 (76%) alleles at *r*^2^ ≥ 0.8 with a mean max *r*^2^ equal to 0.925. The final set of SNPs investigated in this study was rs2723176, rs2723175, rs2723186, rs2723168, rs4364030, rs3811047, rs28947200, rs4392270, rs4849133, and rs2708973.

### Genotyping of *IL-37* SNPs

Genomic DNA was extracted from the buffy coats isolated from patients with HBV using the Gentra Pure Gene kit (Qiagen, Hilden, Germany). Patient and control samples were genotyped for the ten selected SNPs using the 7900 HT Fast Real Time PCR System (Applied Biosystems, Foster City, CA, USA). The reagents used included universal TaqMan master mix, amplifying primers, and probes specific for each SNP and were purchased from Applied Biosystems. For each SNP, one allelic probe was labeled with FAM dye and the other with fluorescent VIC dye. The reaction was performed in a 96-well plate in a total reaction volume of 25 µL using 20 ng of genomic DNA. The TaqMan assay was subsequently read and analyzed by an automated software sequence detection system (SDS, version 2.4.1).

### Statistical analysis

Statistical analysis was performed using the SPSS version 20.0 (SPSS Inc., Chicago, IL, USA) and HaploView version 4.2. The association between the *IL-37* tag SNPs and disease status was expressed in odds ratio (OR) and 95% confidence intervals (CI). A statistically significant level of association was corrected for multiple testing, and only associations less than 0.00125 were considered significant. The SNPs were tested for the Hardy–Weinberg equilibrium (HWE) using Haploview software. A cut-off p-value of 0.05 was set for the HWE, and SNPs were excluded if they did not meet this value. OR values with CI calculated in fixed or random-effects models were used to estimate the strength of the association.

## Results

### Characteristics of the study subjects

Table 1 displays the demographic and clinical details of patients infected with HBV and the control subjects. The analysis shows that older age, male gender, body mass index (BMI), and HBV load were significantly associated with the risk of HBV chronic infection developing into severe liver disease such as cirrhosis and HCC.

**Table 1:** Demographic and clinical characteristics of patients infected with HBV and healthy control subjects.

Variable	Inactive (n = 563)	Active (n = 217)	Cirrhosis (n = 64)	HCC (n = 30)	Healthy Control (n = 599)	Clearance (n = 400)	p-value^a^
Age (yrs.)**	40.65 ± 13.33	36.085 ± 11.76	53.17 ± 12.67	60.034 ± 11.78	30.79 ± 8.93	37.14 ± 10.72	<0.0001
**Sex**							
Male count (%)	380 (67.5%)	174 (80.2%)	51 (79.7%)	29 (96.7%)	567 (94.7%)		<0.0001
Female count (%)	183 (32.5%)	43 (19.8%)	13 (20.3%)	1 (3.3%)	32 (5.3%)		
BMI*	27.70 (24.87–31.73)	27.10 (23.06–30.855)	26.14 (21.815–29.87)	24.055 (21.960–27.44)			0.001
ALT**	58.74 ± 293.82	91.13 ± 108.10	71.32 ± 116.04	80.72 ± 76.44			0.157
HCV Load (Log10)*	2.290 (1.30–3.16)	5.54 (4.50–7.70)	2.77 (1.55–4.922)	3.64 (1.080–5.67)			<0.0001

*Values are expressed as median interquartile range (25th–75th), **Values are expressed as Mean ± SD. p^a^: nonparametric test and one-way ANOVA for continuous data and Chi square test for categorical data. Abbreviations: BMI: Body mass index; ALT: alanine aminotransferase.

### Genotype and allele frequency distributions of *IL-37* polymorphisms associated with HBV infection and clearance

The genotype distribution and allele frequency for *IL-37* polymorphisms between the HBV-infected group and control subjects are summarized in Table 2. The major allele homozygous genotype for each SNP was defined as the reference (Ref) genotype. Our results showed that two SNPs within the *IL-37* gene (rs2723175 and rs2708973) were significantly associated with a higher risk for HBV infection compared to the healthy controls (Table 2). In particular, both SNPs were associated with the highest risk of HBV infection under the dominant model (p < 0.0001, OR = 5.895, 95% CI = 4.216–8.243 and p < 0.0001, OR = 2.768, 95% CI = 1.727–4.438, respectively). Three other SNPs showed suggestive significance at a nominal p-value threshold (rs4849133, rs28947200, and rs2723186). The TT genotype of rs28947200 was associated with a lower number of patients infected with HBV (p = 0.004, OR = 0.462, 95% CI = 0.268–0.796), whereas rs4849133 was related to the risk of HBV infection under both the dominant and recessive models (p = 0.015, OR = 1.419, 95% CI = 1.069–1.885; p = 0.029, OR = 0.552, 95% CI = 0.321–0.947, respectively) compared to healthy controls. The minor allele A of rs2723186 was associated with patients infected with HBV at a nominal p-value, with OR = 1.424, 95% CI = 1.045–1.939 and p-value = 0.024. No significant difference in the genotype and allele distributions of rs2723176, rs2723168, rs4364030, rs3811047, and rs4392270 SNPs was observed in patients infected with HBV compared to the healthy controls (Table 2).

**Table 2:** Comparison of genotypic distributions between patients infected with HBV and healthy controls.

SNPs	Genotype/Allele distribution	Control (n = 599)	%	HBV patients (n = 874)	%	OR (95% C.I.)	χ²	p-value
rs2723176	CC	560	93.49%	794	90.85%	Ref		
	AC	36	6.01%	75	8.58%	1.469 (0.973–2.218)	3.387	0.066
	AA	3	0.50%	5	0.57%	1.175 (0.280–4.939	0.049	1.00
	C	1156	96.49%	1663	95.14%	1.407 (0.965–2.051)	3.172	0.075
	A	42	3.51%	85	4.86%			
	AA + AC vs CC					1.447 (0.972–2.153)	3.340	0.068
	AA vs AC + CC					0.875 (0.208–3.675)	0.033	0.854
rs2723175	GG	554	92.49%	591	67.62%	Ref		
	AG	28	4.67%	238	27.23%	7.968 (5.296–11.987)	127.66	**<0.0001**
	AA	17	2.84%	45	5.15%	2.481 (1.403–4.387)	10.370	**0.001**
	G	1136	94.82%	1420	81.24%	4.232 (3.191–5.613)	114.283	**<0.0001**
	A	62	5.18%	328	18.76%			
	AA + AG vs GG					5.895 (4.216–8.243)	126.976	**<0.0001**
	AA vs AG + GG					0.538 (0.305–0.950)	4.710	0.030
rs2723186	GG	553	92.32%	782	89.47%	Ref		
	AG	28	4.67%	54	6.18%	1.364 (0.853–2.180)	1.691	0.193
	AA	18	3.01%	38	4.35%	1.493 (0.843–2.643)	1.913	0.167
	G	1134	94.66%	1618	92.56%	1.424 (1.045–1.939)	5.071	0.024
	A	64	5.34%	130	7.44%			
	AA + AG vs GG					1.414 (0.977–2.048)	3.392	0.065
	AA vs AG + GG					0.682 (0.385–1.206)	1.752	0.186
rs2723168	AA	19	3.17%	35	4.00%	Ref		
	AG	578	96.49%	834	95.42%	0.783 (0.444–1.383)	0.712	0.399
	GG	2	0.33%	5	0.57%	1.357 (0.240–7.673)	0.120	0.728
	A	616	51.42%	904	51.72%	0.988 (0.853–1.145)	0.025	0.874
	G	582	48.58%	844	48.28%			
	GG + AG vs AA					0.785 (0.445–1.386)	0.698	0.404
	GG vs AG + AA					0.582 (0.113–3.011)	0.426	0.708
rs4364030	CC	292	48.75%	428	48.97%	Ref		
	CG	245	40.90%	357	40.85%	0.994 (0.797–1.239)	0.003	0.958
	GG	62	10.35%	89	10.18%	0.979 (0.686–1.399)	0.013	0.909
	C	829	69.20%	1213	69.39%	0.991 (0.845–1.162)	0.013	0.910
	G	369	30.80%	535	30.61%			
	GG + CG vs CC					0.991 (0.805–1.220)	0.007	0.933
	GG vs CG + CC					1.018 (0.723–1.434)	0.011	0.917
rs3811047	GG	168	28.05%	264	30.21%	Ref		
	AG	278	46.41%	389	44.51%	0.890 (0.695–1.140)	0.850	0.358
	AA	153	25.54%	221	25.29%	0.919 (0.693–1.219)	0.340	0.559
	G	614	51.25%	917	52.46%	0.953 (0.822–1.104)	0.420	0.519
	A	584	48.75%	831	47.54%			
	AA + AG vs GG					0.901 (0.716–1.133)	0.800	0.371
	AA vs AG + GG					1.014 (0.798–1.287)	0.010	0.912
rs28947200	CC	511	85.31%	771	88.22%	Ref		
	CT	55	9.18%	80	9.15%	0.964 (0.672–1.383)	0.040	0.842
	TT	33	5.51%	23	2.63%	0.462 (0.268–0.796)	8.087	0.004
	C	1077	89.90%	1622	92.79%	0.691 (0.533–0.898)	7.739	0.005
	T	121	10.10%	126	7.21%			
	TT + CT vs CC					0.776 (0.571–1.053)	2.660	0.103
	TT vs CT + CC					2.157 (1.254–3.713)	8.047	0.005
rs4392270	GG	555	92.65%	796	91.08%	Ref		
	AG	33	5.51%	54	6.18%	1.141 (0.730–1.783)	0.335	0.562
	AA	11	1.84%	24	2.75%	1.521 (0.739–3.131)	1.316	0.251
	G	1143	95.41%	1646	94.16%	1.288 (0.920–1.803)	2.181	0.139
	A	55	4.59%	102	5.84%			
	AA + AG vs GG					1.236 (0.8841–1.817)	1.166	0.280
	AA vs AG + GG					0.663 (0.322–1.363)	1.268	0.260
rs4849133	TT	513	85.64%	706	80.78%	Ref		
	CT	67	11.19%	119	13.62%	1.291 (0.937–1.778)	2.447	0.118
	CC	19	3.17%	49	5.61%	1.874 (1.090–3.221)	5.312	0.021
	T	1093	91.24%	1531	87.59%	1.475 (1.154–1.886)	9.725	0.002
	C	105	8.76%	217	12.41%			
	CC + CT vs TT					1.419 (1.069–1.885)	5.894	0.015
	CC vs CT + TT					0.552 (0.321–0.947)	4.784	0.029
**rs2708973**	GG	576	96.16%	787	90.05%	Ref		
	AG	9	1.50%	65	7.44%	5.286 (2.611–10.701)	26.342	**<0.0001**
	AA	14	2.34%	22	2.52%	1.150 (0.583–2.267)	0.163	0.686
	G	1161	96.91%	1639	93.76%	2.087 (1.426–3.053)	14.948	**<0.0001**
	A	37	3.09%	109	6.24%			
	AA + AG vs GG					2.768 (1.727–4.438)	19.230	**<0.0001**
	AA vs AG + GG					0.927 (0.470–1.826)	0.050	0.826

Bold indicates significance.

Genotype and allele distribution were also determined in patients infected with HBV and the HBV clearance group due to the natural host immune response. Among the ten *IL-37* polymorphisms, four SNPs (rs2723176, rs2723186, rs4364030 and rs4392270) were significantly associated with a predisposition for HBV clearance compared to patients with chronic HBV infection (Table 3). The A allele of rs2723176, compared to the C allele, showed the highest correlation with HBV clearance (p < 0.0001, OR = 0.517, 95% CI = 0.373–0.716). Under the dominant model, there was a significant association for rs2723176 when comparing chronically infected patients with the clearance group (AA + AC vs CC, p = 0.0002, OR = 0.519, 95% CI = 0.365–0.738). In addition, the AG genotype of rs2723186, compared to the GG genotype (p < 0.0001, OR = 0.369, 95% CI = 0.250–0.543) exhibited a decreased risk of HBV infection. An individual carrying the G minor allele of rs4364030 showed improved viral clearance, with a p-value of 0.0002 and an OR of 0.716 with a 95% CI value of 0.601–0.853. The heterozygous genotype AG of rs4392270 was positively associated with HBV clearance with a p-value < 0.0001, and the A allele was found to be associated with a decreased risk of HBV infection at a nominal p-value level (p = 0.012, OR = 0.667, 95% CI = 0.485–0.918). Similarly, a significant association at a nominal p-value of rs4849133 heterozygous CT genotype was found, when compared to the dominant TT genotype, with p = 0.017 and OR = 0.681. However, two SNPs, rs2723175 and rs28947200, were associated with an increased risk of HBV infection (Table 3). The rs2723175 AG genotype was found to be associated with patients infected with HBV with a p-value < 0.0001 (OR = 10.614 and CI = 6.097–18.480). The rs28947200 CT genotype was associated with the highest risk of HBV infection (p < 0.0001, OR = 20.389, 95% CI = 4.986–83.377) when comparing patients with HBV to the clearance group. No significant difference was found between the HBV clearance group and the HBV infected group in the remaining SNPs (Table 3).

**Table 3:** Comparison of genotypic distributions between patients infected with HBV and the clearance group.

SNPs	Genotype/Allele distribution	Clearance (n = 400)	%	HBV patients (n = 874)	%	OR (95% C.I.)	χ²	p-value
**rs2723176**	CC	335	83.75%	794	90.85%	Ref		
	AC	58	14.50%	75	8.58%	0.546 (0.378–0.786)	10.780	0.001
	AA	7	1.75%	5	0.57%	0.301 (0.095–0.956)	4.650	0.031
	**C**	**728**	**91.00%**	**1663**	**95.14%**	**0.517 (0.373–0.716)**	**16.250**	**<0.0001**
	**A**	**72**	**9.00%**	**85**	**4.86%**			
	**AA + AC vs CC**					**0.519 (0.365–0.738)**	**13.700**	**0.0002**
	AA vs AC + CC					3.096 (0.976–9.814)	4.080	0.043
**rs2723175**	GG	369	92.25%	591	67.62%	Ref		
	**AG**	**14**	**3.50%**	**238**	**27.23%**	**10.614 (6.097–18.480)**	**99.850**	**<0.0001**
	AA	17	4.25%	45	5.15%	1.653 (0.932–2.931)	3.010	0.083
	**G**	**752**	**94.00%**	**1420**	**81.24%**	**3.619 (2.640–4.961)**	**71.080**	**<0.0001**
	**A**	**48**	**6.00%**	**328**	**18.76%**			
	**AA + AG vs GG**					**5.700 (3.848–8.443)**	**89.630**	**<0.0001**
	AA vs AG + GG					0.818 (0.462–1.447)	0.480	0.489
**rs2723186**	GG	331	82.75%	782	89.47%	Ref		
	**AG**	**62**	**15.50%**	**54**	**6.18%**	**0.369 (0.250–0.543)**	**27.150**	**<0.0001**
	AA	7	1.75%	38	4.35%	2.298 (1.016–5.198)	4.210	0.040
	G	724	90.50%	1618	92.56%	0.765 (0.569–1.029)	3.140	0.076
	A	76	9.50%	130	7.44%			
	**AA + AG vs GG**					**0.564 (0.403–0.791)**	**11.240**	**0.0008**
	AA vs AG + GG					0.392 (0.173–0.885)	5.430	0.019
**rs2723168**	AA	4	1.00%	35	4.00%	Ref		
	**AG**	**393**	**98.25%**	**834**	**95.42%**	**0.243 (0.086–0.687)**	**8.320**	**0.004**
	GG	3	0.75%	5	0.57%	0.190 (0.033–1.114)	3.890	0.049
	A	401	50.13%	904	51.72%	0.938 (0.794–1.109)	0.560	0.456
	G	399	49.88%	844	48.28%			
	**GG + AG vs AA**					**0.242 (0.085–0.686)**	**8.350**	**0.004**
	GG vs AG + AA					1.313 (0.312–5.523)	0.140	0.709
**rs4364030**	CC	162	40.50%	428	48.97%	Ref		
	CG	171	42.75%	357	40.85%	0.790 (0.611–1.022)	3.240	0.072
	**GG**	**67**	**16.75%**	**89**	**10.18%**	**0.503 (0.349–0.724)**	**13.920**	**0.0002**
	**C**	**495**	**61.88%**	**1213**	**69.39%**	**0.716 (0.601–0.853)**	**14.040**	**0.0002**
	**G**	**305**	**38.13%**	**535**	**30.61%**			
	GG + CG vs CC					0.709 (0.558–0.901)	7.920	0.005
	GG vs CG + CC					1.313 (0.312–5.523)	0.140	0.709
rs3811047	GG	139	34.75%	264	30.21%	Ref		
	AG	176	44.00%	389	44.51%	1.164 (0.887–1.527)	1.200	0.274
	AA	85	21.25%	221	25.29%	1.369 (0.990–1.892)	3.630	0.057
	G	454	56.75%	917	52.46%	1.189 (1.005–1.407)	4.060	0.044
	A	346	43.25%	831	47.54%			
	AA + AG vs GG					1.231 (0.957–1.582)	2.620	0.106
	AA vs AG + GG					0.797 (0.600–1.059)	2.450	0.118
**rs2894720**	CC	393	98.25%	771	88.22%	Ref		
	CT	2	0.50%	80	9.15%	20.389 (4.986–83.377)	34.710	**<0.0001**
	TT	5	1.25%	23	2.63%	2.345 (0.885–6.215)	3.110	0.078
	C	788	98.50%	1622	92.79%	5.101 (2.805–9.278)	34.910	**<0.0001**
	T	12	1.50%	126	7.21%			
	TT + CT vs CC					7.500 (3.455–16.282)	35.030	**<0.0001**
	TT vs CT + CC					0.468 (0.177–1.241)	2.440	0.119
**rs4392270**	GG	340	85.00%	796	91.08%	Ref		
	AG	52	13.00%	54	6.18%	0.444 (0.297–0.663)	16.420	**<0.0001**
	AA	8	2.00%	24	2.75%	1.281 (0.570–2.881)	0.360	0.548
	G	732	91.50%	1646	94.16%	0.667 (0.485–0.918)	6.260	0.012
	A	68	8.50%	102	5.84%			
	AA + AG vs GG					0.555 (0.388–0.796)	10.490	**0.001**
	AA vs AG + GG					0.723 (0.322–1.623)	0.620	0.429
rs4849133	TT	311	77.75%	706	80.78%	Ref		
	CT	77	19.25%	119	13.62%	0.681 (0.496–0.934)	5.720	0.017
	CC	12	3.00%	49	5.61%	1.799 (0.944–3.429)	3.260	0.071
	T	699	87.38%	1531	87.59%	0.981 (0.762–1.263)	0.020	0.881
	C	101	12.63%	217	12.41%			
	CC + CT vs TT					0.832 (0.622–1.111)	1.560	0.211
	CC vs CT + TT					0.521 (0.274–0.990)	4.090	0.043
rs2708973	GG	364	91.00%	787	90.05%	Ref		
	AG	18	4.50%	65	7.44%	1.670 (0.977–2.856)	3.580	0.059
	AA	18	4.50%	22	2.52%	0.565 (0.300–1.067)	3.170	0.075
	G	746	93.25%	1639	93.76%	0.919 (0.656–1.287)	0.240	0.622
	A	54	6.75%	109	6.24%			
	AA + AG vs GG					1.118 (0.743–1.681)	0.290	0.593
	AA vs AG + GG							

Bold indicates significance.

### Genotype and allele frequency distributions of *IL-37* polymorphisms associated with HBV-related liver diseases

Genotypic and allelic distributions were determined in patients characterized as inactive HBsAg carriers and patients infected with HBV who were considered as active carriers including patients who developed cirrhosis and HCC. Only two SNPs were found to be significantly associated with progression to more severe liver abnormalities at a nominal p-value level; rs4392270 with a p-value of 0.007 (OR = 1.731, 95% CI = 1.159–2.587), and rs4849133 (p-value = 0.021, OR = 1.583, 95% CI = 1.069–2.345). No significant difference in the genotype and allele distributions of the other SNPs was observed between the inactive group compared to the group of patients considered as active carriers, cirrhosis and HCC (Table 4).

**Table 4:** Comparison of genotypic distributions between the inactive group and patients with active HBV, cirrhosis and HCC patients.

SNPs	Genotype/Allele distribution	Inactive (n = 563)	%	Active, cirrhosis and HCC (n = 311)	%	OR (95% C.I.)	χ²	p-value
rs2723176	CC	518	92.01%	276	88.75%	Ref		
	AC	42	7.46%	33	10.61%	1.475 (0.914–2.380)	2.550	0.110
	AA	3	0.53%	2	0.64%	1.251 (0.208–7.533)	0.060	0.806
	C	1078	95.74%	585	94.05%	1.420 (0.914–2.206)	2.460	0.117
	A	48	4.26%	37	5.95%			
	AA + AC vs CC					1.460 (0.917–2.325)	2.560	0.109
	AA vs AC + CC					0.828 (0.138–4.980)	0.040	0.836
rs2723175	GG	374	66.43%	217	69.77%	Ref		
	AG	156	27.71%	82	26.37%	0.906 (0.661–1.242)	0.380	0.539
	AA	33	5.86%	12	3.86%	0.627 (0.317–1.239)	1.830	0.176
	G	904	80.28%	516	82.96%	0.837 (0.648–1.080)	1.880	0.170
	A	222	19.72%	106	17.04%			
	AA + AG vs GG					0.857 (0.636–1.155)	1.020	0.312
	AA vs AG + GG					1.551 (0.789–3.049)	1.650	0.199
rs2723186	GG	509	90.41%	273	87.78%	Ref		
	AG	30	5.33%	24	7.72%	1.492 (0.855–2.602)	2.00	0.157
	AA	24	4.26%	14	4.50%	1.088 (0.554–2.137)	0.060	0.807
	G	1048	93.07%	570	91.64%	1.226 (0.851–1.766)	1.200	0.274
	A	78	6.93%	52	8.36%			
	AA + AG vs GG					1.312 (0.845–2.038)	1.470	0.226
	AA vs AG + GG					0.945 (0.481–1.854)	0.030	0.868
rs2723168	AA	23	4.09%	12	3.86%	Ref		
	AG	537	95.38%	297	95.50%	1.060 (0.520–2.161)	0.030	0.872
	GG	3	0.53%	2	0.64%	1.278 (0.187–8.720)	0.060	0.802
	A	583	51.78%	321	51.61%	1.007 (0.828–1.225)	0.00	0.946
	G	543	48.22%	301	48.39%			
	GG + AG vs AA					1.061 (0.521–2.163)	0.030	0.869
	GG vs AG + AA					0.828 (0.138–4.980)	0.040	0.836
rs4364030	CC	268	47.60%	160	51.45%	Ref		
	CG	241	42.81%	116	37.30%	0.806 (0.600–1.084)	2.040	0.153
	GG	54	9.59%	35	11.25%	1.086 (0.680–1.734)	0.120	0.731
	C	777	69.01%	436	70.10%	0.950 (0.767–1.175)	0.220	0.636
	G	349	30.99%	186	29.90%			
	GG + CG vs CC					0.857 (0.650–1.131)	1.190	0.276
	GG vs CG + CC					0.837 (0.534–1.312)	0.610	0.436
rs3811047	GG	160	28.42%	123	39.55%	Ref		
	AG	267	47.42%	151	48.55%	0.736 (0.540–1.001)	3.820	0.051
	AA	136	24.16%	101	32.48%	0.966 (0.682–1.369)	0.040	0.846
	G	587	52.13%	397	63.83%	0.968 (0.805–1.165)	0.120	0.733
	A	539	47.87%	353	56.75%			
	AA + AG vs GG					0.813 (0.613–1.079)	2.050	0.152
	AA vs AG + GG					0.864 (0.641–1.165)	0.920	0.338
rs28947200	CC	499	88.63%	272	87.46%	Ref		
	CT	49	8.70%	31	9.97%	1.161 (0.723–1.863)	0.380	0.537
	TT	15	2.66%	8	2.57%	0.978 (0.410–2.337)	0.00	0.961
	C	1047	92.98%	575	92.44%	1.083 (0.744–1.576)	0.170	0.676
	T	79	7.02%	47	7.56%			
	TT + CT vs CC					1.118 (0.731–1.709)	0.260	0.606
	TT vs CT + CC					1.037 (0.435–2.473)	0.010	0.935
rs4392270	GG	522	92.72%	274	88.10%	Ref		
	AG	29	5.15%	25	8.04%	1.642 (0.943–2.860)	3.130	0.077
	AA	12	2.13%	12	3.86%	1.905 (0.845–4.297)	2.490	0.115
	G	1073	95.29%	573	92.12%	1.731 (1.159–2.587)	7.330	**0.007**
	A	53	4.71%	49	7.88%			
	AA + AG vs GG					1.719 (1.077–2.745)	5.250	0.022
	AA vs AG + GG					0.543 (0.241–1.223)	2.240	0.135
rs4849133	TT	463	82.24%	243	78.14%	Ref		
	CT	65	11.55%	54	17.36%	1.583 (1.069–2.345)	5.310	0.021
	CC	35	6.22%	14	4.50%	0.762 (0.402–1.444)	0.70	0.404
	T	991	88.01%	540	86.82%	1.115 (0.831–1.495)	0.530	0.468
	C	135	11.99%	82	13.18%			
	CC + CT vs TT					1.296 (0.918–1.829)	2.170	0.141
	CC vs CT + TT					1.406 (0.745–2.656)	1.110	0.291
rs2708973	GG	512	90.94%	275	88.42%	Ref		
	AG	39	6.93%	26	8.36%	1.241 (0.740–2.082)	0.670	0.412
	AA	12	2.13%	10	3.22%	1.552 (0.662–3.637)	1.040	0.309
	G	1063	94.40%	576	92.60%	1.347 (0.909–1.997)	2.220	0.136
	A	63	5.60%	46	7.40%			
	AA + AG vs GG					1.314 (0.837–2.063)	1.520	0.234
	AA vs AG + GG					0.656 (0.280–1.535)	0.960	0.327

Bold indicates significance.

To assess the influence of *IL-37* polymorphism on the risk of HBV-mediated liver disease progression to end-stage liver diseases (liver cirrhosis and/or HCC), the genotype and allelic distributions were analyzed between patients actively infected with HBV and the patients diagnosed with liver cirrhosis with and without HCC. The CT genotype of rs4849133 was found to be nominally significantly associated with progressing to cirrhosis (p = 0.047, OR = 1.986, 95% CI = 1.001–3.940) (Table 5), as well as to cirrhosis with HCC (p = 0.025, OR = 1.990, 95% CI = 1.082–3.658) (Table 6). No significant difference in the genotype and allele distributions of the other SNPs were observed between patients infected with HBV characterized as active carriers and patients diagnosed with liver cirrhosis or liver cirrhosis with HCC.

**Table 5:** Comparison of genotypic distributions between the active group and patients with cirrhosis.

SNPs	Genotype/Allele distribution	Active (n = 217)	%	Cirrhosis (n = 64)	%	OR (95% C.I.)	χ²	p-value
rs2723176	CC	195	89.86%	55	85.94%	Ref		
	AC	21	9.68%	9	14.06%	1.519 (0.658–3.506)	0.970	0.324
	AA	1	0.46%	0	0.00%	1.174 (0.047–29.225)	0.280	0.596
	C	411	94.70%	119	92.97%	1.351 (0.609–2.999)	0.550	0.457
	A	23	5.30%	9	7.03%			
	AA + AC vs CC					1.450 (0.632–3.330)	0.780	0.378
	AA vs AC + CC					0.894 (0.036–22.206)	0.30	0.586
rs2723175	GG	148	68.20%	46	71.88%	Ref		
	AG	61	28.11%	14	21.88%	0.738 (0.378–1.441)	0.790	0.373
	AA	8	3.69%	4	6.25%	1.609 (0.463–5.587)	0.570	0.451
	G	357	82.26%	106	82.81%	0.962 (0.571–1.620)	0.020	0.885
	A	77	17.74%	22	17.19%			
	AA + AG vs GG					0.839 (0.454–1.553)	0.310	0.576
	AA vs AG + GG					0.574 (0.167–1.972)	0.790	0.373
rs2723186	GG	193	88.94%	55	85.94%	Ref		
	AG	15	6.91%	6	9.38%	1.404 (0.520–3.789)	0.450	0.502
	AA	9	4.15%	3	4.69%	1.170 (0.306–4.470)	0.050	0.818
	G	401	92.40%	116	90.63%	1.257 (0.629–2.512)	0.420	0.516
	A	33	7.60%	12	9.38%			
	AA + AG vs GG					1.316 (0.578–2.995)	0.430	0.512
	AA vs AG + GG					0.880 (0.231–3.351)	0.040	0.851
rs2723168	AA	9	4.15%	3	4.69%	Ref		
	AG	207	95.39%	61	95.31%	0.884 (0.232–3.368)	0.030	0.857
	GG	1	0.46%	0	0.00%	0.905 (0.029–27.858)	0.330	0.569
	A	225	51.84%	67	52.34%	0.980 (0.661–1.454)	0.010	0.921
	G	209	48.16%	61	47.66%			
	GG + AG vs AA					0.880 (0.231–3.351)	0.040	0.851
	GG vs AG + AA					0.894 (0.036–22.206)	0.30	0.586
rs4364030	CC	115	53.00%	30	46.88%	Ref		
	CG	81	37.33%	25	39.06%	1.183 (0.648–2.160)	0.30	0.584
	GG	21	9.68%	9	14.06%	1.643 (0.683–3.954)	1.240	0.265
	C	311	71.66%	85	66.41%	1.279 (0.839–1.951)	1.310	0.252
	G	123	28.34%	43	33.59%			
	GG + CG vs CC					1.278 (0.731–2.234)	0.740	0.389
	GG vs CG + CC					0.655 (0.284–1.511)	1.00	0.318
rs3811047	GG	73	33.64%	19	29.69%	Ref		
	AG	84	38.71%	29	45.31%	1.326 (0.687–2.561)	0.710	0.399
	AA	60	27.65%	16	25.00%	1.025 (0.485–2.164)	0.00	0.949
	G	230	53.00%	67	52.34%	1.026 (0.692–1.523)	0.020	0.897
	A	204	47.00%	61	47.66%			
	AA + AG vs GG					1.201 (0.655–2.200)	0.350	0.554
	AA vs AG + GG					1.146 (0.605–2.173)	0.180	0.675
rs28947200	CC	194	89.40%	55	85.94%	Ref		
	CT	17	7.83%	8	12.50%	1.660 (0.680–4.050)	1.260	0.262
	TT	6	2.76%	1	1.56%	0.588 (0.069–4.987)	0.240	0.622
	C	405	93.32%	118	92.19%	1.184 (0.560–2.499)	0.20	0.658
	T	29	6.68%	10	7.81%			
	TT + CT vs CC					1.380 (0.604–3.155)	0.590	0.443
	TT vs CT + CC					1.791 (0.212–15.160)	0.290	0.587
rs4392270	GG	188	86.64%	58	90.63%	Ref		
	AG	18	8.29%	5	7.81%	0.900 (0.320–2.531)	0.040	0.842
	AA	11	5.07%	1	1.56%	0.295 (0.037–2.331)	1.510	0.219
	G	394	90.78%	121	94.53%	0.570 (0.249–1.305)	1.810	0.178
	A	40	9.22%	7	5.47%			
	AA + AG vs GG					0.671 (0.265–1.695)	0.720	0.396
	AA vs AG + GG					3.364 (0.426–26.566)	1.490	0.223
rs4849133	TT	177	81.57%	46	71.88%	Ref		
	CT	31	14.29%	16	25.00%	1.986 (1.001–3.940)	3.950	0.047
	CC	9	4.15%	2	3.13%	0.855 (0.179–4.094)	0.040	0.844
	T	385	88.71%	108	84.38%	1.455 (0.829–2.553)	1.720	0.189
	C	49	11.29%	20	15.63%			
	CC + CT vs TT					1.732 (0.909–3.297)	2.830	0.092
	CC vs CT + TT					1.341 (0.282–6.372)	0.140	0.711
rs2708973	GG	190	87.56%	59	92.19%	Ref		
	AG	20	9.22%	3	4.69%	0.483 (0.139–1.683)	1.360	0.244
	AA	7	3.23%	2	3.13%	0.920 (0.186–4.550)	0.010	0.919
	G	400	92.17%	121	94.53%	0.681 (0.294–1.574)	0.820	0.366
	A	34	7.83%	7	5.47%			
	AA + AG vs GG					0.596 (0.220–1.618)	1.050	0.306
	AA vs AG + GG					1.033 (0.209–5.102)	0.00	0.968

**Table 6:** Comparison of genotypic distributions between the active group and patients with cirrhosis and HCC.

SNPs	Genotype/Allele distribution	Active (n = 217)	%	Cirrhosis + HCC (n = 94)	%	OR (95% C.I.)	χ²	p-value
rs2723176	CC	195	89.86%	81	86.17%	Ref		
	AC	21	9.68%	12	12.77%	1.376 (0.647–2.927)	0.690	0.406
	AA	1	0.46%	1	1.06%	2.407 (0.149–38.956)	0.410	0.523
	C	411	94.70%	174	92.55%	1.438 (0.723–2.860)	1.080	0.298
	A	23	5.30%	14	7.45%			
	AA + AC vs CC					1.423 (0.684–2.961)	0.890	0.344
	AA vs AC + CC					0.431 (0.027–6.957)	0.370	0.541
rs2723175	GG	148	68.20%	69	73.40%	Ref		
	AG	61	28.11%	21	22.34%	0.738 (0.417–1.309)	1.080	0.298
	AA	8	3.69%	4	4.26%	1.072 (0.312–3.683)	0.010	0.911
	G	357	82.26%	159	84.57%	0.846 (0.531–1.348)	0.50	0.480
	A	77	17.74%	29	15.43%			
	AA + AG vs GG					0.777 (0.453–1.333)	0.840	0.359
	AA vs AG + GG					0.861 (0.253–2.933)	0.060	0.811
rs2723186	GG	193	88.94%	80	85.11%	Ref		
	AG	15	6.91%	8	8.51%	1.448 (0.609–3.443)	0.710	0.401
	AA	9	4.15%	5	5.32%	1.340 (0.436–4.124)	0.260	0.608
	G	401	92.40%	168	89.36%	1.366 (0.756–2.470)	1.070	0.300
	A	33	7.60%	18	9.57%			
	AA + AG vs GG					1.407 (0.693–2.859)	0.90	0.343
	AA vs AG + GG					0.770 (0.251–2.363)	0.210	0.647
rs2723168	AA	9	4.15%	3	3.19%	Ref		
	AG	207	95.39%	90	95.74%	1.304 (0.345–4.931)	0.150	0.695
	GG	1	0.46%	1	1.06%	3.00 (0.140–64.262)	0.530	0.468
	A	225	51.84%	96	51.06%	1.032 (0.733–1.453)	0.030	0.858
	G	209	48.16%	92	48.94%			
	GG + AG vs AA					1.312 (0.347–4.961)	0.160	0.688
	GG vs AG + AA					0.431 (0.027–6.957)	0.370	0.541
rs4364030	CC	115	53.00%	45	47.87%	Ref		
	CG	81	37.33%	35	37.23%	1.104 (0.653–1.867)	0.14	0.711
	GG	21	9.68%	14	14.89%	1.704 (0.798–3.639)	1.92	0.166
	C	311	71.66%	125	66.49%	1.274 (0.882–1.841)	1.67	0.196
	G	123	28.34%	63	33.51%			
	GG + CG vs CC					1.228 (0.756–1.993)	0.690	0.406
	GG vs CG + CC					0.612 (0.297–1.264)	1.790	0.181
rs3811047	GG	73	33.64%	31	32.98%	Ref		
	AG	84	38.71%	38	40.43%	1.065 (0.603–1.881)	0.050	0.827
	AA	60	27.65%	25	26.60%	0.981 (0.5 24–1.838)	0.00	0.953
	G	230	53.00%	100	53.19%	0.992 (0.704–1.398)	0.00	0.964
	A	204	47.00%	88	46.81%			
	AA + AG vs GG					1.030 (0.616–1.723)	0.010	0.909
	AA vs AG + GG					1.055 (0.611–1.820)	0.040	0.848
rs28947200	CC	194	89.40%	78	82.98%	Ref		
	CT	17	7.83%	14	14.89%	2.048 (0.963–4.356)	3.580	0.059
	TT	6	2.76%	2	2.13%	0.829 (0.164–4.197)	0.050	0.820
	C	405	93.32%	170	90.43%	1.479 (0.800–2.735)	1.570	0.210
	T	29	6.68%	18	9.57%			
	TT + CT vs CC					1.730 (0.868–3.450)	2.470	0.116
	TT vs CT + CC					1.308 (0.259–6.603)	0.110	0.744
rs4392270	GG	188	86.64%	86	91.49%	Ref		
	AG	18	8.29%	7	7.45%	0.850 (0.342–2.11)	0.120	0.726
	AA	11	5.07%	1	1.06%	0.199 (0.025–1.564)	2.890	0.089
	G	394	90.78%	179	95.21%	0.495 (0.235–1.043)	3.550	0.059
	A	40	9.22%	9	4.79%			
	AA + AG vs GG					0.603 (0.265–1.374)	1.470	0.225
	AA vs AG + GG					4.966 (0.632–39.030)	2.840	0.092
rs4849133	TT	177	81.57%	66	70.21%	Ref		
	CT	31	14.29%	23	24.47%	1.990 (1.082–3.658)	5.010	0.025
	CC	9	4.15%	5	5.32%	1.490 (0.482–4.608)	0.480	0.486
	T	385	88.71%	155	82.45%	1.673 (1.036–2.701)	4.50	0.033
	C	49	11.29%	33	17.55%			
	CC + CT vs TT					1.877 (1.073–3.285)	4.950	0.026
	CC vs CT + TT					0.770 (0.251–2.363)	0.210	0.647
rs2708973	GG	190	87.56%	85	90.43%	Ref		
	AG	20	9.22%	6	6.38%	0.671 (0.260–1.730)	0.690	0.406
	AA	7	3.23%	3	3.19%	0.958 (0.242–3.794)	0.00	0.951
	G	400	92.17%	176	93.62%	0.802 (0.406–1.586)	0.40	0.525
	A	34	7.83%	12	6.38%			
	AA + AG vs GG					0.745 (0.336–1.653)	0.530	0.468
	AA vs AG + GG					1.011 (0.256–3.998)	0.00	0.987

### Haplotype analysis

The haplotype combinations for the *IL-37* polymorphisms and their genotypic distribution in HBV-infected patients and the clearance group were determined. The haplotype containing the C allele of rs4364030, A allele of rs3811047, and C allele of rs2723176 (CAC) (Supplementary Fig. 1) was found to be significantly associated with HBV clearance (p < 0.0001, freq. = 0.402) (Table 7). Moreover, the distribution of two haplotypes (GGC and CAA) with lower frequencies was found to be significantly different when comparing patients infected with HBV to the clearance group (p < 0.0001, freq. = 0.313; p < 0.001, freq. = 0.055, respectively) (Table 7).

**Table 7:** Haplotype frequencies of *IL-37* between the clearance group and patients infected with HBV.

Haplotypes			Freq.	Case, Control Ratio Counts^*^	Case, Control Frequencies^*^	Chi Square	p-value
rs4364030	rs3811047	rs2723176					
C	A	C	0.402	748.1: 999.9, 275.4: 524.6	0.428, 0.344	16.008	**<0.0001**
G	G	C	0.313	503.5: 1244.5, 293.4: 506.6	0.288, 0.367	15.81	**<0.0001**
C	G	C	0.21	383.4: 1364.6, 151.9: 648.1	0.219, 0.190	2.883	0.089
C	A	A	0.055	77.6: 1670.4, 62.1: 737.9	0.044, 0.078	11.724	**<0.001**
G	A	C	0.014	28.0: 1720.0, 7.4: 792.6	0.016, 0.009	1.858	0.173

^*^Case = HBV-Infected patients, Control = Clearance group. Bold indicate significance.

## Discussion

In the absence of an effective anti-HBV treatment^32^, there is an urgent need for predictive genetic tools to characterize patients with a higher susceptibility to HBV infection, clearance and to HBV-mediated liver diseases. Such genetic screening could support improved therapeutic outcomes^32^. It is well-established that host genetic variations are highly important in the development of HCC in HBV-infected patients. Therefore, it is essential to identify biomarkers for high-risk patients for improved management and treatment. Such markers could be useful in predicting tumor aggressiveness, progression and clinical phenotype. Host genetic markers have been identified for colorectal cancer^33^, breast cancer^34^ and other types of cancer^35^.

As immunity plays a pivotal role in the natural course of HBV, the outcome of the infection, and the pathogenesis of liver disease, the genes encoding inflammatory mediators (e.g., TNF-α, TGF-β, and IL-37) may be prospective candidates to predict the progression of HBV-mediated disease severity^36,37^. Here, we investigated the frequency of genetic variants within the *IL-37* gene, a recently discovered immune-suppressive cytokine, and determined the degree of association with HBV infection and different levels of HBV-related pathogenesis progression. More specifically, we were interested in identifying any association between the studied SNPs and spontaneous HBV clearance and/or development of severe forms of HBV-associated disease, such as those involving development of liver complications. Among ten *IL-37* SNPs genotyped, our results revealed that the A allele of rs2723175 was strongly associated with the risk of HBV infection and viral clearance. Additionally, rs4849133 showed a suggestive association with risk for being an active HBsAg carrier and for HBV-mediated end-stage liver disease progression.

Increased risks for HBV infection and HBV-related liver disease are primarily influenced by host factors such as age, gender, BMI, and genetic characteristics^38,39^. In this study, we confirmed the positive influence of these host factors on the increased risk for HBV infection and for HBV-related liver disease. However, on an individual basis, the search for potential predictive genetic risk factors for HBV infection and HBV-mediated end-stage liver disease progression has raised considerable attention in the field of human medical genetics for improved therapeutic management approaches.

In a normal non-infected state, the human liver contains resident antigen-nonspecific immune cells involved in innate immunity, such as natural killer cells, dendritic cells and macrophages named Kupffer cells. The liver also contains cells involved in adaptive immunity including antigen-specific immune T and B cells. Following liver infection with HBV, inflammation occurs due to the production and release of inflammatory cytokines by hepatocytes and immune cells following the binding of the C-terminal domain of HBV core proteins to membrane heparan sulfate on the cell surface^19,40^. It has been reported that IL-37 inhibits various functions, such as antigen presentation^21^, macrophage activation^41^, and cytokine production^42^. Here, among the ten *IL-37* SNPs screened, half show a significant association of susceptibility to HBV infection, including rs2723175, which in our study was found to be strongly disease-associated variants through the heterozygous AG genotype with a dominance of risk allele “A”. Among the ten *IL-37* SNPs analyzed in the Saudi population, three SNPs, rs2723176, rs2723186, rs3811047, have been reported to have a genetic predisposition for auto-immune thyroid disease in the Chinese population^28^. Polymorphisms of other anti-inflammatory cytokines such as IL-10 and IL-4 are also reported to be strongly associated with the outcome of HBV infection^43,44^.

HBV clearance and clinical recovery occur mainly through the induction of effective intrahepatic virus-specific CD4^+^ and CD8^+^ T cells^45^. Thus, the loss of T-cell activity or a decrease in T-cell ability to produce key antiviral and immune stimulatory cytokines shifts from HBV viral clearance toward HBV viral persistence. In comparison with patients undergoing HBV clearance, two *IL-37* polymorphisms (rs2723175 and rs28947200) were strongly associated with HBV infection, suggesting an inhibition of T-cell activity which reinforces the immunosuppressive effects of IL-37. IL-37 has also been demonstrated to inhibit antigen-specific T-cell proliferation^46^. In addition, IL-37 has been demonstrated to be expressed by immunosuppressive regulatory T cells^47^, which are correlated with chronic HBV infection and demonstrated to exert an active influence on HBV clearance^48^. However, six out of ten polymorphisms, including rs4849133, were associated with HBV clearance compared to HBV infection. This strong positive correlation of IL-37 polymorphisms with HBV clearance suggests an inability of *IL-37* genetic variants to inactivate T-cell activity and subsequently offering a protective role of *IL-37* polymorphisms for HBV clearance. In a normal non-infected person, low levels of steady state IL-37 mRNA and protein are found expressed in monocytes, dendritic cells, and plasma cells. However, under inflammatory conditions, *IL-37* gene expression is stimulated with pro-inflammatory cytokines, such as IL-1β, IL-18, TNF-α, IFN-γ, and TGF-β, or Toll-like receptor (TLR) ligands, and downregulated by IL-12, IL-32, and GM-CSF plus IL-4. This immune mechanism suppresses the proinflammatory cytokines IL-1β, IL-1α, IL-6, M-CSF, and GM-CSF but not the anti-inflammatory cytokines IL-10 and IL-1Ra^22^. Thus, a comparative study of *IL-37* genetic variants in patients infected with HBV for IL-37 production, at both the transcript and protein levels, will clarify the role of IL-37 in HBV clearance and persistence.

Globally, inactive HBsAg carriers form the largest group in chronic HBV-infected patients, indicating the tolerogenic status of HBV immunopathogenesis as patients infected with HBV do not display any discernable clinical disease. In contrast to inactive HBsAg carriers, the active carriers contain a high level of serum HBV DNA and circulating serum HBeAg with a high risk for developing liver cirrhosis and HCC. In this study, among the ten genetic variants for the IL-37 gene, only one SNP, rs4849133 with the CT genotype, showed a suggestive association with the risk for active HBsAg carriers compared to patients with inactive HBV infection. These findings are not surprising as the difference between inactive and active carriers based on the viral production of serum HBV DNA copies without involving the host’s innate immune system. However, we previously reported that the haplotype of the CXCR1, a receptor for T-cell chemo-attractant cytokines such as IL-8, named Haplo-2 (AC genotype), was significantly associated with HBsAg carrier status^49^, confirming the important influence of the adaptive T-cell immune system on the HBsAg carrier status for differences in IL-37. Although a previous study has reported that an increased serum IL-37 in patients with chronic HBV infection was positively correlated with liver damage^24^, we only identified the SNP rs4849133 CT genotype as being associated with the susceptibility to end-stage liver disease progression in patients infected with HBV when compared to active carriers infected with HBV. Recently, IL-37 has been described to exhibit anti-tumor activity through chemo-attraction of CD57^+^ natural killer (NK) cells, inhibiting HCC development^23^. Thus, patients infected with HBV harboring *IL-37* SNP rs4849133 might fail in the production of active IL-37 protein, which may explain the increased risk for HCC progression. Furthermore, no mutation within *IL-37* gene has been found in all the HCC cases described in The Cancer Genome Atlas - Liver Hepatocellular Carcinoma project (TCGA-LIHC). However, a modulation of the HCC tumor immune milieu including the depletion of neutrophils and activated macrophages, main sources of IL-37, have been recently reported in a TCGA-LIHC subset of HBV/HCV-infected patients only^50^; which could also explain the decrease of IL-37 production contributing to HCC development and progression.

This study is limited by the fact that the sample sizes in the cirrhosis and HCC groups were small. Also, this study does not include in the final analysis some important factors, such as treatment protocol, treatment outcome and duration of the infection, well known to impact the course of HBV infection. Similarly, survival analyses were not conducted in our study due the cross-sectional feature of this study. Additional studies are required to include such analyses and validate these results. In conclusion, these findings suggest that *IL-37* polymorphisms may not only be implicated in the development of HCC but may also be involved in HBV infection and in determining different clinical outcomes of HBV infection, including active chronic HBV infection and low viremic “inactive” HBsAg carrier status.

## Supplementary information

Supplementary file
