# Supplementary material for: Association between IL-37 gene polymorphisms and risk of HBV-related liver disease in a Saudi Arabian population
Source: Sci Rep. 2019 May 9;9:7123. doi: 10.1038/s41598-019-42808-4 (PMC6509272; doi:10.1038/s41598-019-42808-4)
Supplement: Supplementary file 1 — Supplementary file [file 41598_2019_42808_MOESM1_ESM.docx]

**Association between *IL-37* gene polymorphisms and risk of HBV-related liver disease in a Saudi Arabian population**

Mashael R. Al-Anazi^1^, Sabine Matou-Nasri^2^, Arwa A. Al-Qahtani^3^, Jahad Alghamdi^2^, Ayman A. Abdo^4,9^, Faisal M. Sanai^5,9^, Waleed K. Al-Hamoudi^4,9^, Khalid A. Alswat^4,9^, Hamad I. Al-Ashgar^6^, Mohammed Q. Khan^6^, Ali Albenmousa^7^, Monis B. Shamsi^8^, Salah K. Alanazi^1^, Damian Dela Cruz^1^, Marie Fe F. Bohol^1^, Mohammed N. Al-Ahdal^1,10^, Ahmed A. Al-Qahtani^1,10,*^

^1^Department of Infection and Immunity, Research Center, King Faisal Specialist Hospital & Research Center, Riyadh, Saudi Arabia.

^2^Medical Genomics Research Department, King Abdullah International Medical Research Center, Ministry of National Guard, Riyadh, Saudi Arabia.

^3^Department of Family Medicine, Prince Mohammed Bin Abdul Aziz Hospital, Riyadh, Saudi Arabia.

^4^Section of Gastroenterology, Department of Medicine, College of Medicine, King Saud University, Riyadh, Saudi Arabia.

^5^Gastroenterology Unit, Department of Medicine, King Abdulaziz Medical City, Jeddah, Saudi Arabia.

^6^Gastroenterology Unit, Department of Medicine, King Faisal Specialist Hospital & Research Center, Riyadh, Saudi Arabia.

^7^Department of Gastroenterology, Prince Sultan Medical Military City, Riyadh, Saudi Arabia.

^8^Centre for Genetics and Inherited Diseases, College of Medicine, Taibah University, Madinah, Saudi Arabia.

^9^Liver Disease Research Center, King Saud University, Riyadh, Saudi Arabia.

^10^Department of Microbiology and Immunology, Alfaisal University School of Medicine, Riyadh, Saudi Arabia.

Correspondence to: Ahmed A. Al-Qahtani, PhD

Email: aqahtani@kfshrc.edu.sa


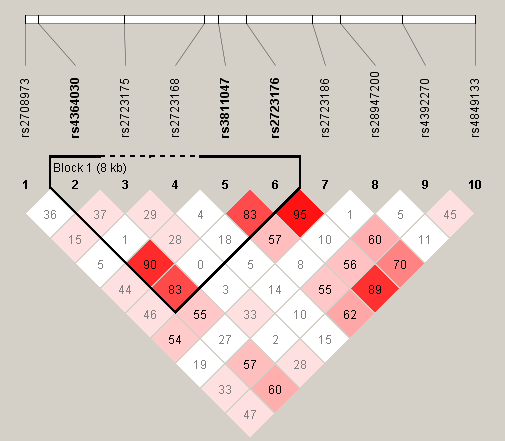


Supplementary Figure 1. The haplotype block structure showing the IL-37 SNPs selected in the analysis between clearance group and HBV infected patients.

| Supplementary Table 1: Minor allele frequency of the selected SNPs from the 1000 Genome project | | | | | | | | | |
| --- | --- | --- | --- | --- | --- | --- | --- | --- | --- |
| SNP | Location | Allele | 1000 Genome Project Allele Frequency | | | | | | Saudi* |
|  |  |  | AF | AFR | AMR | EAS | EUR | SAS |  |
| rs28947200 | 2:112918606 | T | 0.008 | 0.0303 | 0 | 0 | 0 | 0 | 0.101 |
| rs2723168 | 2:112913313 | A | 0.0761 | 0.1755 | 0.0591 | 0.001 | 0.0835 | 0.0235 | 0.0534 |
| rs2723175 | 2:112910176 | A | 0.0845 | 0.208 | 0.0562 | 0.001 | 0.0845 | 0.0235 | 0.0518 |
| rs4849133 | 2:112923864 | C | 0.217 | 0.524 | 0.08 | 0.135 | 0.082 | 0.12 | 0.0876 |
| rs4364030 | 2:112906832 | G | 0.336 | 0.146 | 0.34 | 0.464 | 0.434 | 0.36 | 0.308 |
| rs3811047 | 2:112913833 | A | 0.3826 | 0.7625 | 0.1945 | 0.1885 | 0.2992 | 0.2883 | 0.4875 |
| rs2723186 | 2:112917503 | A | 0.1422 | 0.3517 | 0.0259 | 0.1329 | 0.002 | 0.0951 | 0.5142 |
| rs4392270 | 2:112921052 | A | 0.1144 | 0.2519 | 0.0173 | 0.1329 | 0.002 | 0.0941 | 0.0459 |
| rs2723176 | 2:112914932 | A | 0.0908 | 0.3525 | 0.0245 | 0.1329 | 0.002 | 0.0941 | 0.0306 |
| rs2708973 | 2:112906296 | A | 0.0401 | 0.1437 | 0.0144 | 0 | 0.001 | 0 | 0.0309 |
| * Allele frequency in this study using healthy control as the reference.  AF, Global allele frequency; AFR, African population; AMR, American; EAS, East Asian; EUR, European; SAS, South Asian; SNP, Single Nucleotide Polymorphism. | | | | | | | | | |
